# Extra Limites

**Published:** 1821-03-01

**Authors:** 


### EXTRA LIMITES;

OR

### DEPARTMENT FOR DISQUISITIONS, DEFENCES, EXPLANATIONS, &c. &c.*

### I.

### *Dr. Jenner's Circular Letter to the Profession.*

SIR,

PRESUMING that you are conversant with the practice of Vaccine Inoculation according to the instructions which I have formerly published, and that you may have seen, in addition to my general observations, those which I have since made and promulgated, respecting the "Varieties and Modifications of the Vaccine Pustule, occasioned by an

---

* As this department is entirely beyond the standard limits of the Review, the conditions of insertion may be seen in No. III, p. 551.

herpetic and other eruptive states of the skin," I take the liberty of requesting to be informed, whether the observations acquired in your own practice coincide with mine? That is to say, whether the Vaccine Vesicles, under these contingent circumstances, go through their course with the same regularity, as when the skin is free from diseases of this description?

*Secondly*. Whether, on the other hand, such individuals are more liable to resist the legitimate action of Vaccine Lymph when inserted into the arms, than those who are free from such eruptive affections?

*Thirdly*. Whether you have met with cases of Small-Pox, or what has been termed the Varioloid Disease, after Vaccination; and if so, whether, in such cases, you ascertained those deviations at the time of Vaccination, in the progress of the pustules on the arms, which I have described as liable to take place when the skin is affected with herpetic and other eruptions?

As you may not have the paper before you, to which I here allude, nor the short series which followed it, I will point out the periods of their publication, and where they are to be found. The first was published in the Medical and Physical Journal, No. 66, for August 1804, and gives an outline of the subject, of some extent. It points out the fact, that a single serous blotch upon the skin, existing during the progress of the Vaccine Vesicles on the arms, may occasion such irregularity and deviation from correctness, that Vaccination, under such circumstances, cannot be perfectly depended upon.

I have found abrasions of the cuticle to produce the same effect; such, for example, as we find in the nurseries of the opulent, as well as the cottages of the poor, behind the ears, and upon many other parts where the cuticle is thin. Happily we find no irregularity in the Vaccine Vesicle in an uncontaminated skin; but we find it if the skin is beset with these herpetic blotches, or even simple serous oozings from an abraded cuticle. It is not to be considered as of less consequence when occupying a small space; a speck behind the ear which might be covered by a split pea, being capable of disordering the progress of the Vaccine Vesicle. Dandriffe may be considered as a malady of this class, the incrustation on the scalp being formed from excoriation beneath; and however slight, for there is every gradation between a thin scurfy layer of a dirt-looking substance, or even patches of this thin crust, and Tinea itself. However, fortunately for the safety of the Vaccine Practice, and fortunately too for the ease of the practitioner, all these affections of the skin may be removed with very little trouble.* Sore eyelids are also impediments to constitutional Vaccination.

The second paper relating to this subject was given by the late Dr. Willan, in answer to the following interrogatory, addressed to me by himself:† "What are the changes produced in the vesicle, when a person is affected, during Vaccination, with the Shingles, the Vesicular Ringworm, or Impetigo?"

To this question I made a full, and, I believe, a satisfactory reply. Its purport will be shewn by quoting a few sentences from it. "To answer this question in its fullest extent, would lead me through a wide field of observation, which I mean to go over at a future time; but the following answer may probably convey to you as much information upon the subject as you may now require." "Vaccination, under the circumstances you mention, usually produces a striking deviation from the perfect character of the Vaccine vesicle at some period or other of its progress, but more frequently in its early, than in its declining stages; indeed, it is commonly perceptible in a day or two after inoculation. It would be difficult, perhaps impossible, without the aid of drawings, to give a correct description of the varieties which an herpetic state of the skin is capable of producing, from those trifling deviations which prove no impediment to the Vaccine security, up to that point of imperfection in the vesicle which affords no security at all. Perhaps I commit an error in saying "*no security at all*," for it strikes me, that the constitution loses its susceptibility of Small-Pox contagion, and its capability of producing the disease in its perfect and ordinary state, in proportion to the degree of perfection which the Vaccine vesicle has put on in its progress, and that the Small-Pox, taken subsequently, is modified accordingly.‡

---

* The most effectual application which I know for subduing these cuticular diseases, that produce impediment, is the Unguentum Hydrargyri Nitratis, as much lowered with Unguentum Cetacei, or any other bland ointment, as the irritability of the subject may require. The Dandriffe demands a double process—the *first* consists in removing the incrustation, the *second* in subduing the oozing. There are skins that will not well bear unctuous applications; the desiccative lotions may then be made use of two or three times a-day; such as those prepared with the sulphate of zinc or superacetate of lead, &c.

† It was published in the year 1806, in his Treatise on Vaccine Inoculation.

‡ Further observation has confirmed this opinion, and has also developed much other curious matter respecting the spontaneous blending of the herpetic with the vaccine fluid, through the medium of the constitution, when under the influence of Herpes.

When no deviation takes place in the ordinary course of the Vaccine vesicles, or when it is inconsiderable, the Herpetic blotches or vesicles, of whatever kind they may be, often assume (sometimes as early as the third or fourth day after the insertion of the Vaccine fluid) a new character, not unlike the Vaccine, and, keeping pace in their progress with the vesicles on the arm, die away with them, leaving the skin smooth."

These two papers comprehend, first, the simple fact of important deviations being produced by diseases in pre-occupation of the skin; and, secondly, a general account of the characters of these deviations, and their differing degrees of influence upon the Vaccine protection.

Some further observations were published by Dr. Wilson Philip, M. D. of Worcester, now of London, in an Appendix to his Work on Febrile Diseases, who requested some information from me on this interesting subject. This letter goes more into detail than the former, though its purport is the same—namely, to guard the practitioner against the insidious influence of a diseased skin, when he vaccinates. It will be an object of future consideration, to enter more generally into the minutiæ of this subject; but a sketch like this does not afford scope for the completion of such a design. Let me advise every practitioner, not to confine his cautions, nor to narrow my meaning, to one class of eruptive affections. In short, every disease of the skin, which may be called *serous*, or one that sends out a fluid capable of conversion into a scab, has the power of exerting this modifying and counteracting influence; and I have also seen purulent fluids exert a similar influence in producing deviations. If I was asked what were the other actual impediments to perfect Vaccination, as a general answer I should say, that I scarcely know any other except spurious matter,* or impediments too obvious to require my naming them here, such as deranging the Vaccine vesicle in its progress, by incautiously robbing it of its contents, or producing a new action by external violence.

I have the honour to be, Sir,

Your obedient humble Servant,

EDW. JENNER.

*Berkeley*, 28*th January*, 1821.

---

* I am happy to see that these interruptions are now discovered in Germany, as appears in Professor Hufeland's Journal for June 1819; an extract of which is given in the London Medical Repository, vol. xiv. p. 502.

In addition, see Bateman's Synopsis of Cutaneous Diseases, pp. 222, 223. Cross's History of the Variolous Epidemic at Norwich, 1820, pp. 60, *et seq.* 196, and 288. I was lately puzzled to find the cause of irregularity in a Vaccine vesicle, the skin being free from any apparent eruption; upon minute enquiry I discovered a whitlow on the thumb, in which suppuration had taken place.

### II.

*Dispensary for Diseases of the Skin, Great Marlborough Street, under the Patronage of His Royal Highness the Duke of York, and other distinguished personages.*

*Consulting Physicians*, Dr. Bateman and Sir Matthew Tierney, Bt.
*Physicians*, Dr. Thomson, Dr. Yetch, and Dr. Ashburner.
*Surgeons*, Mr. Carpue, and Mr. Wadd.

We are gratified to find, from a Report of the Committee to the Patrons of this very meritorious Institution, that between the 1st January, 1820, and the 1st January, 1821, 245 cases of cutaneous complaints were treated, most of them being cured, and almost all relieved. "The eminent relief and comfort experienced by many indigent families in various parts of this extensive metropolis, from the use of the baths and medicines gratuitously supplied to them, afford unequivocal proof of the utility of the Institution. Small as is the number of patients admitted, compared with similar institutions of other capitals, yet the *variety* of cutaneous diseases, we understand, was such, that almost every one described in Willan's splendid work has been seen here.

"One, among many remarkable and instructive cases the Medical Officers wish to bring under your notice.—It was an inveterate instance of the pompholyx, which had resisted the various treatments of several hospitals.—It occurred in a young girl; her sufferings were not to be described; but by the use of the bath, and some internal medicines, she now enjoys a comparative degree of health and comfort; and her tender age renders it extremely probable that the cure may be as permanent as it has been satisfactory." *Report of Committee.*

Many of the most distinguished medical characters in the profession (among whom we need only instance a Halford and a Baillie) have patronized the Institution, and we conceive that it is only necessary to make its existence known to a liberal public, in order to secure a still greater support. To the profession we think it is a most desirable thing, that an Institution, directed by medical officers of such respectability, should be at hand to assist the remedial treatment of a class of diseases, many of which have hitherto proved opprobria to the healing art.

### EXTRA LIMITES.

*(Continued from page 784.)*

### III.

### *Dr. Granville's Reply to a Review in the Journal of Science.*

THE accompanying reply to an article contained in Mr. Brande's Journal for January 1821, on the subject of my work on Prussic Acid, was sent to that gentleman, through the hands of a friend, for insertion in his next Number. The result of this application will be better understood by a perusal of the following laconic correspondence.

*Saville-row, Wednesday Evening,*

*7th of February.*

*Dear Sir,*

*I send you a reply to the article contained in your last Number, purporting to be a review of my work on the Prussic Acid. Dr. Hutchinson, a particular friend of mine, has been kind enough to undertake to deliver it into your own hands, to prevent mistakes; and he is instructed to request you will have the goodness to name an hour in the evening of to-morrow, when he may call for your decision, as to whether you will admit, or not, the said reply for insertion in your next number for April. In case of your declining to insert it—a circumstance which I am far from contemplating—I have requested my friend to bring back the manuscript, without any further comment.*

*I have the honour to be, your humble servant,*
*A. B. GRANVILLE.*

*W. T. Brande, Esq. F.R.S. &c. &c.*

Two days afterwards, Mr. Brande returned me the manuscript, with a short note, of which the following is the beginning.

*Thursday (not delivered till Friday night.)*

"*My dear Sir,*

"*You must surely be quizzing me, to suppose that I should insert the inclosed, &c.*

"*I am always yours, faithfully,*

*W. T. BRANDE.*"

"*Dr. Granville, Saville-row.*"

How far, by the refusal of an act of justice and impartiality demanded of him, and by the language in which that refusal is conveyed, Mr. Brande has or not identified himself with my reviewer—and thereby rendered himself obnoxious to all and each of the charges I have brought forward and *proved* against the latter, I leave the reader to decide. It is enough for me to observe that Mr. Brande's conduct, as the editor of the Quarterly Journal, in this affair, is to me a matter of great astonishment; and that, as a member of the Royal Institution, I shall take the earliest opportunity of protesting, either at a general meeting, or to the board of managers, against that Society's lending its name to a journal in which an attack, involving matters of personal consideration, is admitted, and the reply, shewing the injustice, and unfairness of that attack, rejected.

Under these circumstances I avail myself of the facility, which the EXTRA LIMITES department of the Medico-Chirurgical Review has liberally opened to all parties, of giving publicity to my reply to the review in question.

A. B. GRANVILLE.

SAVILLE ROW,
*Friday*, 9*th February*, 1821.

*A Reply to an Article inserted in the 20th Number of the Quarterly Journal of Science, edited at the Royal Institution—purporting to be a Review of Dr. Granville's Treatise on the internal Use of the Hydrocyanic Acid.*

In a Letter to W. T. BRANDE, Esq. F.R.S.
The EDITOR.

DEAR SIR,

You will readily remember, that when you informed me of having received a "severe" critique on my work on Prussic Acid for insertion in your Journal, of which, however, you assured me that no use would be made, if I no longer entertained the opinion I expressed in that work respecting the acid prepared at Apothecaries' Hall—I instantly replied, that you were welcome to admit and insert the article in question, if you thought proper: for as my opinion of the acid prepared at the Hall, at the specific period at which I was writing, had been formed upon such ocular and experimental demonstration as would have warranted still stronger expressions of disapprobation on my part—I could not tamely surrender my humble judgment to the terror of any review of my book, however severe. The only concession I claimed in return was, that any reply that I might think it necessary to write to the article you mentioned, should be equally honored with insertion in your Journal—and to the justice of this claim you readily acceded.*

In order to bring these circumstances more particularly to your memory, it will be well to mention that the conversation

* The reader will have seen, from his reply to my letter given above, in what manner Mr. Brande has redeemed his implied pledge.

above referred to took place on the evening of the 14th of December, 1820, at Somerset House, a short time before the meeting of the Royal Society; and that it was again repeated by you, in a more cursory, yet impressive, manner, immediately after the Society had adjourned, as we were both in the act of leaving the meeting-room.

Had the review been "severe," yet just, correct, and candid, I should have preserved a becoming silence, and endeavoured to profit by the admonitions bestowed on me, however harshly; but as it possesses none of the latter qualities, I can scarcely be expected to suffer it to pass unnoticed.

When I state that the review is the reverse of being just, correct, and candid, I am advancing what, I trust, I shall fully prove to yourself and the public; and this circumstance induces me to declare, *in limine*, that, although by designating that review as "severe," you implicitly acknowledged that you had read it; yet, from my acquaintance with your character and urbanity of manners, I am free to assume that you had not paid any very particular attention to the structure of that article, and the assertions it comprises; or it would never have appeared in your Journal, to which I am proud in having been one of the earliest, and certainly not the least zealous of its contributors.

My observations, therefore, can, in no way whatever, apply to you as the editor of that Journal, except where it is explicitly so stated; but are directed to the writer, of whom the letter O stands as the representative.

To render my reply as perspicuous, and as concise as the nature of the subject will admit, I shall, whenever I have an opportunity, adopt the form of *positive answer* to the assertions of the reviewer—rather than argumentative discussion, for which I have neither the necessary talent, nor any very particular inclination. No answer or reply will be given, to which I am not prepared to append a proof in support of its meaning—this being, in my opinion, the surest mode of conducting a defence.

The circumstances which induced the reviewer to notice my work are stated by him to be these:—

1. "The first part of it (the work) affects scientific arrangement; and the subject of which it treats *was first brought before the British public*, in this Journal."

2. "We wish to point out *an error or two* into which the Doctor has fallen."

3. "And to advertise him of *two or three samples of bad taste* which have probably escaped his notice."

To which I then reply, seriatim.

Reply 1. The purely scientific part of the subject of Hydrocyanic Acid was NOT first brought before the British public in the Quarterly Journal of Science; neither was its application to the purposes of medicine first adverted to in that Journal.

PROOFS. A masterly account of the discoveries and investigations of Gay Lussac on that subject was given in the Annals of Philosophy for December, 1815, at which epoch the Quarterly Journal was NOT in existence! And a paper *specifically written for the British public*, respecting the use of Prussic Acid as a medicine, was inserted in the Medical Repository, two years and a half before the subject was noticed in the Quarterly Journal.

COROLLARY. O's first asseveration, therefore, is "incorrect."

Reply 2. Of the one or two errors into which I am said to have fallen by the reviewer, *one only* is mentioned, after all, by him in the course of his critique at page 402, respecting the specific gravity of prussic acid; and that is NOT an error of the author, but a typographical fault.

PROOF. See the *errata corrige* prefixed to my work, in which that fault is actually rectified.

COROLLARY. O's first accusation against me, therefore, is "unjust."

Reply 3. Of the two or three samples of bad taste of which O was anxious to "advertise me," one only is brought forward by him in his review—and that one sample of bad taste is only made to appear as such by artfully coupling together two short garbled quotations from my book.

PROOF. The anecdote related in my book, from which the quotations of the reviewer are taken, relates to a matter of fact, which he has not dared to repeat; or what he has called "bad taste," would have appeared to be "plain truth."

COROLLARY. O's conduct, therefore, is "wanting in candour."

After sketching a short and rapid account of the history of prussic acid, taken in some instances, *verbatim*, though without acknowledging it, from my work, the reviewer proceeds to assert, page 401, that:—

1. I have adverted to the history of that substance *superficially*.

2. That I have given the different processes for preparing the acid, *without sufficient remarks upon their principles*.

3. That I have passed judgment upon the merits of those processes, *not always tempered with mercy*.

To which assertions I beg to apply the following answers:—

Answer 1. The chemical history of Prussic Acid is NOT adverted to superficially in my treatise. It is more fully given, than in many recent works on chemistry. It is a hundred times more extended than that substituted by the reviewer for the edification of his readers.

PROOFS. My chemical history of Prussic Acid begins from the discovery of Prussian blue, and terminates with the latest researches respecting the component parts and real nature of the acid itself, by Gay Lussac, whose notions and atomic theories are fully given; while the intermediate epochs of this interesting history are duly noticed, the labours of several eminent chemists, particularly those of Morveau, Scheele, Berthollet, &c. as well as those of M. Porrett, in this country, are mentioned; and continued reference made throughout the section, as well as at the conclusion of it, to various works in which the subjest has been treated in the most satisfactory manner. The historical account above alluded to occupies *twenty-four* printed pages of my work; while not one-third of that space, in the most approved modern works on chemistry, has been dedicated to that subject, excepting in the laborious and classical system of chemistry by Dr. Thomson. The chemical history of the same substance, substituted by the reviewer himself, occupies just *twenty-nine lines*.

COROLLARY. O's assertion, therefore, respecting my "superficiality," is incorrect.

Answer 2. It is NOT true, that I have given the different processes for preparing the acid "without any sufficient remarks upon their principles."

PROOFS. My description of Scheele's process, written with what perspicuity I could master, is followed up by a complete *rationale* of the various steps of that process, which had been ambiguously interpreted by others! Of Vauquelin's process I observed that its simplicity would render any remark of mine upon it an act of supererogation—and on the third, or Magendie's process, consisting in the mere dilution of the concentrated acid with water, I made enough remarks to attract the notice of the reviewer, who has grounded upon them a whole and long paragraph concerning their pretended incorrectness!

COROLLARY. O's assertion, therefore, is again, in this instance, "unjust."

Answer 3. The judgment I passed on the different processes, is so far from being "not tempered with mercy," that in two cases it is given in a strain of eulogium—and in the third no judgment is given at all!

PROOFS. Speaking of that of Scheele, I state, "By this method the acid is obtained at an uniform degree of concentration;" and again, "this acid is perfectly good for the purpose of practice;" while, on the subject of that of Vauquelin, I affirm, "that the acid prepared according to his method is of a proper strength for medicinal purposes."

COROLLARY. O's assertion, therefore, in this case, is again "wanting in candour."

And here I cannot help observing to you, my dear Sir, that this advocate for mercy so far forgets his own precepts, that throughout the review of my work sentiments are uttered and expressions used that are very distantly allied, indeed, to that heaven-born virtue. As a proof of this assertion, I need only mention that the very processes of two such eminent chemists as Scheele and Vauquelin, which he affects to defend from my unmerciful judgment, are, by him, dismissed in the most peremptory language of condemnation—the one as furnishing an acid of "variable composition," the other for being "extremely objectionable."

Speaking of the very curious but difficult branch of chemical inquiry, respecting the formation of Prussic Acid by the combination of animal matter, contained in the third section of my book, the reviewer says, that "I should either not have meddled with it, or given a clear epitome of what is known upon the subject." In answer to which I have to observe,

1st. That I have NOT meddled with the subject which is still involved in absolute obscurity, any further than by repeating an uncontroverted fact, which, instead of being quoted in a garbled manner, should have been fairly transcribed by the reviewer; and 2dly, That the whole of what is known on the subject of the formation of Prussic Acid by the combination of animal matter. *is* detailed in the said section, although that *all* be but little, and betray "a poverty in the land," as the reviewer poetically expresses it. And I challenge him to point out any book on chemistry in which more, or even as much, is to be found on that subject, that is not conjectural.

PROOFS. The very paragraph with which the section in question of my work begins, and which the reviewer imperfectly quotes, as unintelligible, is given in the identical language of Berthollet, from whose *Essai de Statique Chimique* it was collected—neither Thomson, nor Murray, nor Henry, nor Klaproth, nor Orfila, nor Lagrange, no—nor even yourself, in your manual, have alluded to the inquiry in question in the slightest degree: so that the "epitome of what is known," may be well comprehended in the two pages that I have dedicated to that subject.

COROLLARY. The reviewer, therefore, is on the present occasion, as on all the preceding ones, wanting in "justice" as well as "candour."

But I have done more:—I have given an extract from my notes, taken while attending a course of lectures on animal chemistry, delivered by Vauquelin, in which an endeavour is made to place the above subject in a somewhat clearer light. Yet what is the conduct of O on this occasion? With the same *nonchalance* with which he had, just before, talked of the *unintelligibility* of two paragraphs borrowed from Berthollet and Thenard, he assures his readers, that the extract from Vauquelin's lectures is not a tittle more intelligible; and dismisses it without any quotation in support of his affirmation. Now, as I mean, throughout this letter, to substantiate by PROOFS what I advance on the score of O's candour and justice; and as I hesitate not to assert, that the charge of unintelligibility against Vauquelin is as gross a defection from truth, as that which characterizes the charge brought against his process for preparing the hydrocyanic acid, I shall beg leave to quote, once more, the passage in question, in order that the reader may judge, whether or not it be *unintelligible*. "When animal substances are exposed to heat with a mixture of alcalies—hydrogene, carburetted, and carbonic gas are obtained, besides a *residuum*, which, if washed in water, will be found to contain prussic acid. The alcali, therefore, seems necessary to form the prussic acid, by attracting together the principles of which it is constituted," &c.* If this be unintelligible, then plain language is not capable of expressing common ideas; but if, on the contrary, the passage be found perfectly comprehensible, and such, indeed, as will be met in substance, in the works of most men of eminence who have written on the same subject, then the conclusion regarding the reviewer's candour is unavoidable.

The reviewer's charge, at page 406, of my having unnecessarily separated the account of the physical properties of the prussic acid, from its chemical history and preparation, is of too trivial a nature, and absurd, to need refutation. Every book on chemistry is full of examples of such practice; and Gay Lussac himself has followed no other method in his admirable essay on hydrocyanic acid.

In the succeeding paragraph of his critique, relating to the physiological experiments made with the *pure hydrocyanic acid* by several authors and myself, the reviewer remarks, that "as Mr. Brodie's investigations *upon this subject*, are the most satisfactory that have hitherto been made; and, as they are not even alluded to by me, he shall decline troubling his readers with those I have detailed." To which, this is my reply:

1. Mr. Brodie NEVER made any experiment with the *pure hydrocyanic acid.*
2. Previous and subsequently to Mr. Brodie's investigation respecting the action of various poisons on animals, Coullon, Emmert, Magendie, and others had and have instituted experiments with the pure hydrocyanic acid, or with substances containing it, not

* It is not a little curious that one of the most important works on Chemical Science, written in the English language, should contain a passage nearly similar in import to the above, on the subject of Prussic Acid. Having described the process of heating blood and alcalies, to procure the acid—the author proceeds to give the following explanatioo of that process. "This process consists essentially of two operations, one the impregnation of the alcali with that peculiar principle contained in the blood, which gives the power of striking a blue colour with iron, and is called the prussic acid," &c. *Vide Aikin's Dictionary of Chemistry, Art. Prussian Blue.*

only upon animals, but upon the human system, which, in a work of practical utility, and not simply of philosophical speculation, could not but be preferred to every other experiment.

Either, therefore, O knew all this; and in such a case, where is candour and truth in concealing it?—or he knew it not; and in that case, it was his duty, ere he undertook to criticise the book, to have made himself master of its subject.

The eighth section of the work relates to the means of detecting prussic acid, and preventing its poisonous effects: "in neither of which, says the candid and just reviewer, do we remark any thing either very new or very important;" but respecting which I must beg leave to ask him two questions.

1. Is it not *very important* to determine the symptoms of poisoning by this acid, and to ascertain the best means for counteracting its deleterious effects? These objects have been accomplished, as far as they could be, in the said eighth section.

2. Is it not *very important* to be acquainted with the means of detecting the presence of prussic acid, particularly in cases of death from that substance? And have these means, or the mode of conducting the investigation, been pointed out to the public before the appearance of my work, by any chemist, English or foreign? Is not that new, which is not to be found elsewhere? Is any thing of the kind contained in the works of Fourcroy, Chaptal, Thenard, Thomson, Murray, Orfila, Henry, Children, or even in your own manual of chemistry? No.

Then what becomes of the justice, correctness, candour, and I may now add, the knowledge, in these matters, of the reviewer? Perhaps some evidence of all these qualities is to be found in the remaining part of the Review, relating to the most important as well as to the largest portion of my work, which is dismissed in ten lines and a half!

I am now arrived at that part of my reply, upon which I enter with feelings of great reluctance; because it alike involves charges of a heavy nature against the reviewer; and obliges me, from a sense of what is due to truth, publicly to deny the correctness of opinions said to be your own.

My charges against the reviewer are, 1. Misrepresentation, or concealment of facts. 2. Ignorance of the subject on which he has undertaken to pronounce. 3. Unworthy insinuations against the author, whose book he reviews. Each and all of which charges, in pursuance of the plan I have followed throughout this reply, I shall proceed to substantiate by positive proofs; leaving, however, to the public, the task of drawing, in this instance, the corollaries that must necessarily follow.

The first charge, or that of misrepresentation or concealment of facts, is supported by the following evidence:—1. The reviewer tells his readers that the formula of Dr. Magendie for diluting Gay Lussac's acid, is not given in my book (p. 402); whereas the *fact* is, that at page 20 of my Treatise *I have inserted that formula* thus: "Dr. Magendie dilutes the concentrated acid of Gay Lussac with six times its volume, or eight times and a half its weight of distilled water."—2. The reviewer, in the same paragraph, informs his readers that the number 9.20583 is quoted by me as the "medium density" of Magendie's diluted acid; whereas the truth is, that I distinctly used the word *weight*, meaning the absolute weight, and not the "*medium density*" of a mixture of 8.5 of water and 0.70583 of concentrated acid.—3. The reviewer says, I have "fallen into some sad errors respecting the specific gravity of the pure acid," and merrily and triumphantly quotes a typographical fault by which I am made absurdly to state the specific gravity of the acid to be 70.583; whereas in the *errata*, which every candid reviewer would have turned to on seeing such an absurd mistake, the printer's error is actually rectified, and the specific gravity correctly given thus, .70583.—4. The reviewer states, "that the doctor insinuates, though he must know better, that the acid sold at Apothecaries' Hall is *always* turbid, yellowish, and *impure*." This is NOT true. The *Doctor* never expressed such an insinuation; nor did he use the two words, "always" and "impure;" which the reviewer, with utter disregard to propriety, has attributed to him, and has even marked in italics. The following is the only passage in which I passed any degree of condemnation on the acid prepared at Apothecaries' Hall: "I know, besides, that the acid thus prepared is of a turbid yellowish colour, instead of being colourless and transparent, and that it deposits a considerable sediment; both which circumstances *seem* greatly to militate against its *purity*."

The second charge against the reviewer, or that of ignorance of the subject on which he has undertaken to pronounce, is thus substantiated. 1. The reviewer states at page 400, that he is "not quite clear" whether Gay Lussac gave the name of *cyanogene* to the base of hydrocyanic acid "because it burns with a blueish purple flame, or because it is essential to the production of Prussian blue;" whereas, Gay Lussac is positive as to the latter reason; and every chemist acquainted with the subject knows it to be so.—2. The reviewer asserts, that the acid obtained by Scheele's method, "is of variable composition, because Prussian blue is not always of equable purity:" but this is not true in practice. Mr. Garden has prepared prussic acid according to this method for the last three years and a half, with an invariable precision of result and equality of strength, which, to judge of its constant specific gravity, is stronger than that prepared at Apothecaries' Hall.—3. The reviewer has acknowledged his inability of obtaining *pure* prussic acid by Vauquelin's method, which, he asserts to have tried *frequently*. But the want of success in his case must be ascribed to his ignorance in chemical manipulations, *as you well know;* for you *have seen* pure prussic acid prepared according to that method.—4. The reviewer says he cannot understand the following passage of my work, containing the well known hydrodynamic axiom, that "the weight of fluids is equal to their volumes multiplied by their densities." I can only observe, in reply, that if he is a stranger to mathematical language he has only to refer to Dr. Young's lectures on Natural Philosophy, or Biot's, or Brisson's, or any other author's work on the same subject, in all of which that very axiom is mentioned and explained. In the instance above alluded to, the passage was translated *verbatim* from a note in Dr. Magendie's pamphlet on the subject of which I was commenting.—5. The reviewer, making himself quite merry, and rioting in the pleasures of the discovery of the "Doctor's sad errors," asserts that the specific gravity of a mixture of six volumes of water at 1. and one volume of acid at 0.70583, such as Magendie employs, *must* be 0.9900: whereas, any person acquainted with the common rules of alligation, would know that $\frac{6\times 1+0.7853}{7}=0.9579$. To which I add that such is nearly the actual specific gravity of the mixture in question, ascertained by repeated experiments—the "increase of density resulting from the mixture of the pure acid with water" not being so "great" as the reviewer boldly asserts.

The third charge against O, namely, that of throwing out unworthy and unwarrantable insinuations against me, will be best substantiated by a quotation of his own expressions. "We should have conceived it more decorous on the part of Dr. Granville, finding the above preparation objectionable, as he has asserted it to be, to have stated the objections to the Apothecaries' Company, *instead of publishing their process with a view to depreciate it, and to employ it as a vehicle of a puff-oblique in favour of the Doctor's chemist, Mr. Garden.*" I despise too much the individual, who, without the slightest degree of evidence in support of them, can assume and publish two such inferences, and direct them against the moral rectitude of an author—to be disposed to take any other notice of the above disgraceful insinuations against my character, than to express my utter astonishment, that you should have suffered it to appear in your Journal! Did you know of any thing in my conduct during the six or eight years of our acquaintance; or any passage in my work which could have led you to admit, for one instant, the propriety of the aspersions of my reviewer? The only passage that relates to Mr. Garden the chemist, and which is contained in the same paragraph of my work, which seems to have given such mortal offence to O, is this: "I have not had an opportunity of trying this acid (the apothecaries) as I am satisfied with that which Mr. Garden prepares for my patients." Is *truth*, then, synonimous with *puff-oblique* in the moral lexicon of my reviewer?—As to its being or not "*more decorous*" for me to have stated the objections I entertained against their acid, to the Apothecaries' Company themselves, instead of publishing those objections; I have yet to learn that that worshipful body have any claim to the services of any physician. Let them look to their own business; and see that their own officers do their duty. O gives a sort of manifesto in his review, from the Apothecaries' Company, in which they declare, that they "*have no secrets;*" if so, why should he feel sore at my having published their process of preparing the hydrocyanic acid? and how, I would furthermore ask him, can the publication of such a process, "*depreciate it*," as he rather aukwardly states, if the process be inherently perfect?

A few more words on the subject of certain opinions ascribed by the reviewer to yourself; and on the defence set up by that gentleman in favor of the Apothecaries' acid of 1819 and 1820, and I conclude.

You are said in the Review, to have reported to the Laboratory Committee of the Apothecaries' Society that, "having tried the methods of Scheele and Vauquelin, you found them uncertain in their products, and more especially in the latter case, the specific gravity of Vauquelin's acid *always* exceeding that of distilled water." In opposition to which opinions, I assert, 1. That Scheele's method, with common precaution, yields, by far, the most equable and the purest acid for medicinal purposes, of a specific gravity inferior to that quoted by you as the density of the acid prepared according to your own formula, and which is stated to be 0.995, although I have found it to be as high as 0.998 in two specimens procured at the Hall a few weeks back—whereas, 0.993 is the invariable density of Scheels's acid at an uniform temperature. 2. That by Vauquelin's method, prussic acid, possessing all the requisite physical and medicinal properties, colourless, transparent, and of the specific gravity of 0.998, and, consequently, *under the standard density* of distilled water, can be procured—is annually procured to a large amount by the French chemists, who sell no other—and has been procured by me at two different times, since your assertion to the contrary has been published by the reviewer. You have not, I suppose, forgot, that, on the evening of the 18th ultimo, I shewed you, at the table of the Royal Society, a specimen of the acid so prepared by me, as well as another, prepared, according to Scheele's process, by Mr. Garden, which you admitted to be "as good specimens as could be desired, and the purity of which, to judge of their specific gravity marked on the phials containing them, seemed to be quite unobjectionable." It was on that same occasion, that I remarked to you, that the accuracy of Vauquelin was too well established, to allow us for a moment to suppose, that he would have recommended a process which, according to your expressed opinion, "is uncertain in its products," and the specific gravity of which "is *always* greater than that of distilled water." To the justice of this remark you then assented. The specimens alluded to were shewn that same evening, and in the same place, to Dr. Holland and Messrs. Philips and Faraday, to whom, as well as to you, I exhibited two other specimens of the acid, procured at Apothecaries' Hall in 1819 and 1820. One of these, presented a fluid of a dark-brown colour—turbid, when shaken—but transparent when suffered to rest so as to give time for a copious blackish sediment to subside: the other offered a fluid of a muddy colour and appearance, though by no means so striking as in the former case. From these two specimens, obtained at two different periods from the Hall, I then declared to you, and to the other gentlemen abovementioned, what I again repeat on the present occasion, that the description of the Apothecaries' acid, contained in the second edition of my work, was taken; and I may, I think, call upon you to say, whether under such circumstances, I should not have been justified in passing a *severer* judgment upon the merits of that acid, than that which my reviewer has qualified by the expression, "not tempered with mercy;" and to which I omitted to advert in another part of this reply, because I considered the present as a fitter opportunity for so doing.

The fact is, that both you and the reviewer—the one verbally, in conversation with me—the other in writing, at page 404 of his Review, have admitted the justice of my remark as to the appearances of the acid prepared at Apothecaries' Hall; and it remains only for me to say, in conclusion, that if my reviewer can assert, as he has done at page 404, that "the occasional yellowness and turbid appearance of that acid is *rather an indication of its purity than otherwise*," his logic will certainly fail to convince his readers, as it failed to convince myself. If, however, by the admission that the acid is occasionally yellow and turbid, and presents, moreover, a sediment, its decomposition is also admitted (which the reviewer has virtually done); then a preparation liable to such fatal objections, ought not to have been defended as a "very uniform and very pure product;" nor sold as *pure prussic acid* by the Apothecaries' Company, to supply "the occasional demand" for that article, as they have unquestionably done in the case of the two specimens in my possession, which were nearly as objectionable in their appearance when first procured, as they are now, when "age" and "purity," says the reviewer, are to be considered as the two causes of those objectionable appearances. For the information and caution of every medical practitioner, I publicly exhibited, last night, at a meeting of the Medico-Chirurgical Society, the two latter specimens, as well as those alluded to in the course of this letter; which have been prepared agreeably to Scheele's and Vauquelin's methods—and will shew them to any other member of the profession, who may be desirous of inspecting them.*

As to the tone and style, generally, in which O's review is written, I can only assent to the judgment passed upon them, in a scientific circle, by one of the first philosophers in the country;† to whose opinion both you and I must readily bow—which judgment, given in the presence of two or three other gentlemen, but addressed to me in particular, went to condemn both as equally derogatory from the dignity of a reviewer, and injurious to the journal in which such a Review has been admitted.

I have the honour to be your humble servant,

A. B. GRANVILLE.

*Saville-row,*
*Wednesday, 7th Feb.* 1821.

---

* The relative value of the acid prepared by Mr. Garden, and at the Hall, according to Mr. Brande's formula, will be further illustrated by the following facts. A pupil of St. Bartholomew's assured me the other day, that the Apothecaries' acid had been administered to patients in that hospital, in doses of *twenty-four* drops at a time, without the slightest obvious effect. Mr. Travers mentioned to the Medico-Chirurgical Society, a few nights since, that one of the servants of St. Thomas's Hospital, having inadvertently swallowed a mixture containing about eighteen drops of prussic acid, prepared by Mr. Garden, which he mistook for an aperient draught, fell down on the floor as if shot by a cannon-ball! and continued ill for some days.

† This last paragraph has undergone a trifling verbal alteration, since the manuscript was submitted to Mr. Brande, and rejected—in consequence of that gentleman having erroneously interpreted its original meaning, in his note to me, and also with a view to avoid any such misinterpretation.

